# Validity and Reliability of a Commercially-Available Velocity and Power Testing Device

**DOI:** 10.3390/sports6040170

**Published:** 2018-12-10

**Authors:** Andrew T. Askow, Jason D. Stone, Daniel J. Arndts, Adam C. King, Shiho Goto, Joseph P. Hannon, J. Craig Garrison, James M. Bothwell, Phil E. Esposito, Andrew R. Jagim, Margaret T. Jones, Will Jennings, Jonathan M. Oliver

**Affiliations:** 1 The Sport Science Center at Texas Christian University, Department of Kinesiology, Texas Christian University, Fort Worth, TX 76129, USA; a.askow@tcu.edu (A.T.A.); j.stone@tcu.edu (J.D.S.); d.arndts@tcu.edu (D.J.A.); a.king@tcu.edu (A.C.K.); p.esposito@tcu.edu (P.E.E.); will.jennings28@tcu.edu (W.J.); 2 Texas Health Sports Medicine, Fort Worth, TX 76132, USA; shihogoto@texashealth.org (S.G.); josephhannon@texashealth.org (J.P.H.); jamesgarrison@texashealth.org (J.C.G.); jamesbothwell@texashealth.org (J.M.B.); 3 Mayo Clinic Health Systems, Division of Family Medicie, Onalaska, WI 54650, USA; andrew.jagim@gmail.com; 4 Frank Pettrone Center for Sports Performance, George Mason University, Fairfax, VA 22030 USA; mjones15@gmu.edu

**Keywords:** GymAware, Qualysis, velocity-based training, force, velocity, power

## Abstract

Given the relationship between explosive-type training and power adaptation, tracking movement velocity has become popular. However, unlike previous variables, tracking velocity necessitates the use of a valid and reliable tool to monitor adaptation over time. Therefore, the primary purpose of this research was to assess the validity and reliability of a commercially-available linear position transducer (LPT). Nine resistance-trained men completed four sessions consisting of a single set of barbell back squat to volitional failure at 75% or 90% one-repetition maximum. Kinetic and kinematic data were captured for each repetition by the LPT and a 3-dimensional motion capture system and bipedal force platforms. In total, 357 instances of data from both systems were analyzed using intraclass correlations (ICC), effect size estimates, and standard error of measurement. Overall, the LPT yielded excellent ICCs (all ≥0.94) and small/trivial differences (*d* < 0.60). When categorized by median values, ICCs remained high (all ≥0.89) and differences remained small or trivial with the exception of high peak velocities (*d* = −1.46). Together, these data indicate that the commercially-available LPT is a valid and reliable measure for kinetic and kinematic variables of interest with the exception of high peak velocities.

## 1. Introduction

The ability to generate power is a necessary aspect of performance in sport. Therefore, one of the goals of a well-structured training plan should be to augment maximal power production and speed in athletes. For years, coaches and researchers have explored a number of training methods in which to achieve this goal including various periodization schemes, exercise types, volume loads, and relative intensities. Recently, movement velocity has become a popular method for assessing neuromuscular demand and resultant adaptations to training [1,2,3,4]. Much of this popularity stems from the assertion that training specificity should be achieved by maximizing movement velocity in order to promote optimal power adaptation following a training period [5]. However, unlike previous methods of maximizing power adaptations, the use of this type of training necessitates a tool that is capable of measuring movement velocity.

One method of quantification is through the use of a motion capture camera system whereby reflective markers are placed on the body and barbell in order to quantify displacement-time data. In conjunction with a force platform, kinetic and kinematic data can be accurately calculated. However, motion capture camera systems are expensive and impractical outside of a laboratory or clinical setting. As a result, linear position transducers are gaining popularity due to the lower cost, ease of use, and portability. Linear position transducers use a wired tether that attaches to a person or piece of equipment in order to capture displacement-time data. Through differentiation, these data are transformed into estimates of peak and average force, power, and velocity.

In an athletic setting, small changes in power production or velocity can have an impactful effect in competition [6]; therefore, accuracy of tools used to measure kinetic and kinematic data is critical for the successful implementation of velocity-based training. While some authors report that linear position transducers are a viable option that satisfies the accuracy requirements of an athletic setting [1], others question the validity and reliability of the systems [7]. Therefore, the aim of this investigation was to assess the validity and reliability of a commercially-available linear position transducer compared to a motion-capture camera system with a force platform in the barbell back squat as a means to measure peak and average force, velocity, and power. The barbell back squat exercise was chosen for this investigation due to its broad use as a fundamental exercise for power development in an athletic setting [8,9,10]. Due to the direct acquisition of position-time, but not force data, we hypothesized that the system would be valid and reliable for velocity variables but would fail to meet validity and reliability standards for force and power variables.

## 2. Materials and Methods

### 2.1. Experimental Approach to the Problem

A repeated-measures, crossover design was utilized in order to assess the reliability and validity of the linear position transducer. For the initial visit, participants were consented and familiarized with the experimental equipment and procedures then completed one-repetition maximum (1 RM) testing in the back squat exercise. Following 72 h of rest, participants returned to the laboratory for four experimental sessions, each separated by at least 48 h. In these trials, participants completed four random-order bouts of back squat (2 bouts at 75% 1 RM and 2 bouts at 90% 1 RM) to volitional fatigue. Kinetic and kinematic data were collected throughout via motion capture and integrated bipedal force platforms and a commercially-available linear position transducer.

### 2.2. Participants

Nine (n = 9) resistance-trained men (24.3 ± 5.6 yrs; 1.74 ± 0.1 m; 82.5 ± 9.6 kg; 13.5% ± 6.8% body fat; 152.4 ± 19.4 kg 1 RM_bs_; 1.9 ± 0.2 1 RM:body mass (BM) ratio) volunteered to participate in this study. While nine participants completed this study, data from these individuals were pooled for analysis such that a total of 357 instances of data (with the exception of average velocity and power where the number of available data points was 346) from both systems were analyzed to assess validity and reliability. Participants were required to be between the ages of 18 and 35, possess a 1 RM to body mass ratio of at least 1.5, and have at least two years of resistance training experience utilizing the back squat. Further, reported use of nutritional supplement or ergogenic aids within the last twelve months, lower extremity injury within the last year, or lower body surgery within the last three years resulted in exclusion. The aforementioned procedures were carried out in accordance with the Declaration of Helsinki and approved by the Institutional Review Board. Written informed consent was obtained from all subjects prior to enrollment.

### 2.3. Familiarization and 1 RM Determination

Upon arrival at the laboratory, participants’ height and body mass were measured to the nearest 0.5 cm and 0.2 kg, respectively, using a portable stadiometer (Seca, Chino, CA, USA) and self-calibrating digital scale (Seca, Chino, CA, USA). Participants then had their body composition determined from skinfolds using previously established methods [11]. Briefly, the thickness of each skinfold was measured at seven sites on the body at least two times. The thickness of each skinfold was measured using Lange^®^ skin fold calipers. Body density was subsequently estimated [11] and used to calculate body fat percentage using the Siri equation [12]. Following body composition testing, participants completed a standardized dynamic warm-up followed by 1 RM determination in the back squat using a previously established protocol [13]. All 1 RMs were determined within five maximal attempts.

### 2.4. Experimental Trials

At least 72 h following 1 RM assessment, participants returned to the laboratory having refrained from any lower body exercise for at least 72 h and any activities outside of daily living for 48 h prior. Upon arrival, participants completed the same dynamic warm-up used in the 1 RM testing session followed by the commencement of a barbell back squat warm-up. The barbell warm-up consisted of one set of five repetitions (1 × 5) at 40% 1 RM and 1 × 5 at 60% 1 RM separated by two minutes of rest for the 75% condition and the same warm-up with an additional set of three repetitions at 80% 1 RM in the 90% condition. Following two minutes of rest, participants completed one set to volitional failure at either 75% 1 RM or 90% 1 RM on four separate visits (two visits per intensity, randomly assigned) separated by 48 h. Before the beginning of each set, participants were instructed to perform the concentric portion of each repetition “as explosively as possible.” Subjects were instructed to wear the same attire and footwear for each session.

### 2.5. Kinetic and Kinematic Data Acquisition and Processing

During all experimental trials, kinetic and kinematic data were collected concurrently using a commercially-available linear position transducer (GymAware, Kinetic Performance Technology, Canberra, Australia; GYM) and an eight-camera motion capture system (Qualysis, Goteborg, Sweden; QUAL) sampling at 120 Hz in conjunction with a bilateral force platform (AMTI, Watertown, MA, USA) sampling at 1200 Hz. For QUAL, reflective markers placed on each end of the barbell were used to compute average (AV) and peak barbell velocity (PV) in the sagittal plane during the concentric phase of each repetition. The three-dimensional marker position and kinetic data were imported to Visual3D (C-Motion Inc., Germantown, MD, USA) software and filtered using a second order low-pass Butterworth filter with a cut-off frequency at 12 Hz. Utilizing an event threshold approach (Visual 3D), sagittal plane knee joint kinematics were referenced to define the start and end of the descent (squat start to maximum knee flexion) and ascent (maximum knee flexion to squat end) phases of individual repetitions. Total ground reaction force during the ascent was calculated by summing ground reaction forces from each force platform. Average and peak power output (PP and AP) were subsequently calculated by multiplying average and peak total ground reaction force (PF and AF) by AV and PV, respectively, during the ascent phase.

GYM employs a wire tether attached to the end of a barbell to determine the displacement of a barbell. GYM employs a variable rate sampling frequency. Using this method of sampling, displacement >600 microns in distance are recorded at a resolution of 35 microseconds. Data are subsequently down sampled and timestamped at 20 ms time points [14] to yield displacement-time data. These data are subsequently transformed into peak and average force, velocity, and power through the process of differentiation. The first differentiation of these data yields velocity (PV and AV) and the second yields acceleration. Multiplication of these acceleration data by user-inputted system mass (summation of barbell load and participant mass) results in force output (PF and AF). Finally, the product of force and velocity data results yields power output (PP and AP). These data are automatically calculated and transmitted via Bluetooth™ to a tablet (iPad, Apple Inc., Cupertino, CA, USA).

### 2.6. Statistical Analyses

Overall validity and reliability analyses were determined by combining a total of 357 instances in which data were available from both QUAL and GYM (except for AV and AP where only 346 instances were analyzed). In order to determine whether GYM is susceptible to higher error at high or low velocities, power outputs, or force outputs, a sub-analysis was conducted by dividing data into high and low groups at the median for each respective output (mean and peak force, velocity, and power). Subsequently, intraclass correlations (ICCs) were calculated and used in conjunction with effect size (Cohen’s *d*; *d*) estimates to assess the validity of GYM compared to QUAL. Further, Bland–Altman plots, with limits of agreement defined as the mean difference ± 1.96 SD of the difference, were employed to assess the level of concordance between GYM and QUAL [15]. Finally, standard error measurement (SEM) was calculated to further evaluate the reliability of GYM. Based on previous work in this area, GYM was deemed highly valid if there was good agreement between tools (ICC > 0.76) and a trivial or small effect size (*d* < 0.60) according to the Hopkins modified Cohen scale (<0.20, trivial; 0.20–0.60, small; 0.60–1.20, moderate; 1.20–2.0, large; 2.0–4.0, very large; >4.0, extremely large) [14,16,17]. Further, ICCs were classified using the following criteria: excellent (ICC = 0.91–1.00), good (ICC = 0.76–0.90), moderate (ICC = 0.51–0.75), and poor (ICC = 0.00–0.50) [18]. Data are presented as mean (95% CI) where applicable.

## 3. Results

### 3.1. Overall Validity and Reliability

**Figure 1:**
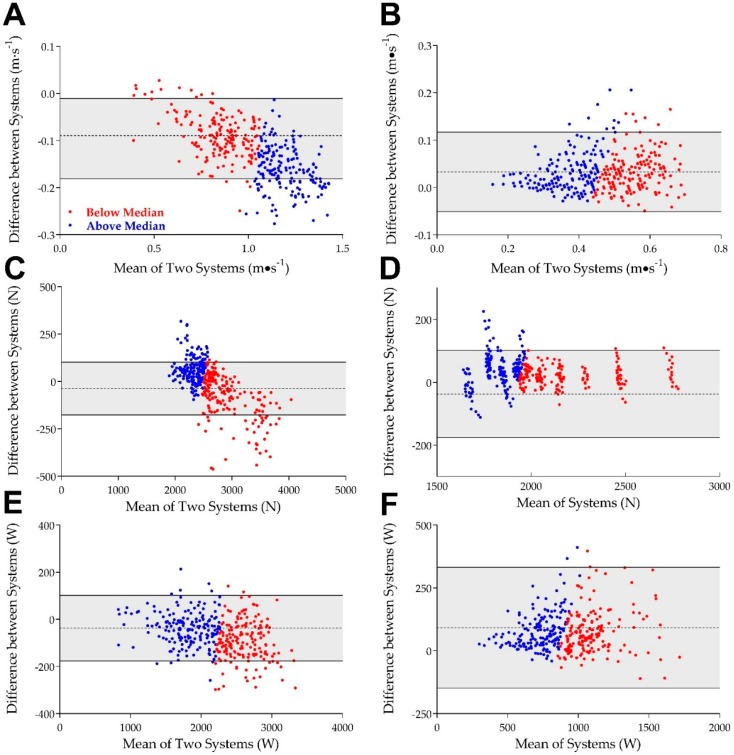
Bland–Altman plots for peak velocity (**A**), average velocity (**B**), peak force (**C**), average force (**D**), peak power (**E**), and average power (**F**) for data below the median (blue circles) and above the median (red circles). Shaded region represents the area between the 95% limits of agreement (solid black lines). The single dashed line represents mean bias for each variable of interest.

Mean bias, ICCs, SEE, and Cohen’s *d* for differences between GYM and QUAL are presented in Table 1. Overall ICCs (consistency) between GYM and QUAL revealed excellent reliability (all *p* <0.001) for PV, AV, PP, AP, PF, and AF (see Table 1). Analysis of Bland–Altman plots revealed that GYM overestimates (mean bias [95% CI]) AV, AP, and AF by 0.03 (0.01, 0.05) m·s^−1^ (*d* = 0.28; see Figure 1B), 75.6 (38.9, 112.3) W (*d* = 0.31; see Figure 1F), and 29. 7 (−8.6, 68.0) N (*d* = 0.11; see Figure 1D), respectively and underestimates PV, PP, and PF by −0.13 (−0.16, −0.09) m·s^−1^ (*d* = −0.57; see Figure 1A), −65.5 (−138.9, 8.0) W (*d* = −0.13; see Figure 1E), and −11.48 (−74.4, 51.4) N (*d* = −0.03; see Figure 1C), respectively.

### 3.2. Analysis of High and Low Values

Intraclass correlation analysis of values above and below the median for each variable revealed excellent reliability both above and below the median for PV, PF, AF, PP, and AP (ICCs all ≥0.91; see Table 1). For AV, GYM had excellent reliability below the median (ICC = 0.914) and good reliability above the median (ICC = 0.894). Further, a large negative bias in values above the median was observed for PV as measured by GYM above the median (bias = −0.16, *d* = −1.46).

## 4. Discussion

The primary aim of this investigation was to assess the validity and reliability of GYM to accurately quantify mean and peak force, velocity, and power. Although uniform standards for the determination of validity and reliability of an instrument have not been determined, previous literature suggests that ICCs above 0.75 and effect size estimates below 0.60 (small or trivial) may be considered reliable [19,20] and valid [14], respectively. In the current investigation, although GYM demonstrated modest underestimations for PV (mean difference = −11.6%) and overestimations for AV (mean difference = 7.1%) and AP (mean difference = 8.6%), differences were deemed small or trivial (see Table 1). Further, ICCs for all outputs were excellent (all ≥0.91). In support of our original hypothesis, these data indicate that GYM is a reliable and valid for measurement of PV and AV. Further, in contrast to our original hypothesis, GYM is valid and reliable for measurement of PF, AF, PP, and AP.

As a supplement to the primary aim, we also sought to determine whether GYM maintained validity and reliability at outputs above and below the median. When fractionated this way, GYM largely underestimated PV at high velocities whereas the difference below median values, low velocities, was small (see Table 1). This was further reflected in ICC values for GYM and QUAL below and above the median value for PV (0.975 and 0.953, respectively). From a practical standpoint, the underestimation at high velocities may pose a problem for practitioners seeking to quantify velocity during explosive-type movements. However, ICCs and effect size estimates for all other variables suggest that GYM is still reliable, both above and below the median.

Although the validity and reliability of GYM has been reported elsewhere [14,21,22,23,24], to the authors’ knowledge, only one other study has assessed the validity and reliability of GYM using an integrated 3-dimensional motion capture system and dual force platforms [25]. In that study, participants performed three separate sessions consisting of three repetitions each of back squat, bench press, and deadlift at a single load (85% 1 RM). Given the limited number of repetitions completed and the use of a single load, that study likely failed to assess GYM across the functional range of values. However, in agreement with current findings, those authors reported excellent correlations (R^2^ ≥ 0.91) between GYM and 3-dimensional motion capture for PV, AV, PF, and AF during those lifts with the exception of AV in the deadlift which the authors postulated was due to differential sampling frequency between GYM and the criterion measure.

Though work comparing GYM to motion capture systems is limited, several authors have sought to assess the validity and reliability of GYM compared to other tools [14,21,22,23,24]. One such study [14] compared GYM to a laboratory-based kinetic and kinematic assessment system consisting of four linear position transducers in conjunction with a force plate to directly acquire ground reaction forces. Similar to the current investigation, the authors assessed GYM across its functional range by utilizing different barbell loads in the back squat (20%, 40%, 60%, 80%, and 100% of 1 RM) and reported validity and reliability for GYM across the entire load–velocity and load–force spectrum. In contrast to current findings, GYM failed to meet validity criterion for PP and AP at very light loads (20% and 40% 1 RM; *d* > 0.60). It is interesting to note that, although GYM failed to meet the threshold for validity at light loads for PP and AP, those authors reported excellent validity for PV at light loads, a finding that is in contrast with the current investigation.

Though those results are conflicting, single LPT systems, such as GYM, have previously been reported to be susceptible to slight variations in horizontal or vertical displacement [7]. Since single LPTs, unlike criterion measures, do not directly acquire ground reaction force data through the use of a force platform, these systems rely on the differentiation of force–time data to yield force and, thus, power. The process of differentiation can amplify any noise in the signal due to those variations in displacement leading to a magnification of the error [21]. Similar to Dorrell and colleagues [25], the current investigation likely allayed this error by utilizing high external resistance, reducing the instance of horizontal barbell motion [25]. However, the possibility of this error should be noted, and special care should be taken to mitigate these errors if kinetic outputs are of interest to the user.

The current study employed only a single exercise and relatively high external loads. While our data represent a broad range of outputs, the loads employed likely mitigate some of the error observed in low external load exercise. Further, the barbell back squat is a relatively linear movement. Since the degree of error observed is seemingly related to the degree of horizontal displacement when using a single LPT system, these factors represent a limitation of the current investigation and may preclude application of our data to other exercises and loads which have a greater instance of horizontal movement. However, given the frequent use of the back squat exercise in a practical setting [8,9,10] and the large range of outputs observed in this investigation, these data provide valuable information in determining the suitability of GYM in assessing kinetic and kinematic outputs.

## 5. Conclusions

The present study suggests that GYM is a viable alternative to laboratory-based systems for practitioners seeking to monitor training and assess adaptation in the back squat except for at high peak velocities where practitioners should be cautious in the interpretation of data. Overall, despite the lack of a direct force measurement, GYM is a valid and reliable tool to quantify peak and average force, velocity, and power across its range of functional values.

## Figures and Tables

**Table 1:** Mean bias, intra-class correlations (ICC), standard error of estimate (SEE), and Cohen’s *d* for the GymAware power tool. PV = peak velocity; AV = average velocity; PF = peak force; AF = average force; PP = peak power; AP = average power.

PV (m·s^−1^)	Mean Bias	Cohen’s *d*	ICC	SEE
Below Median	−0.09 (−0.12, −0.06)	−0.59 (−0.80, −0.38)	0.975 (0.967, 0.982)	0.04 (0.04, 0.05)
Above Median	−0.16 (−0.18, −0.14)	−1.46 (−1.69, −1.22)	0.953 (0.936, 0.965)	0.05 (0.04, 0.05)
All Values	−0.12 (−0.16, −0.09)	−0.57 (−0.72, −0.42)	0.982 (0.978, 0.985)	0.05 (0.04, 0.05)
**AV (m·s^−1^)**				
Below Median	0.03 (0.02, 0.05)	0.43 (0.22, 0.64)	0.914 (0.884, 0.937)	0.03 (0.03, 0.04)
Above Median	0.03 (0.02, 0.04)	0.48 (0.26, 0.69)	0.894 (0.957, 0.921)	0.03 (0.03, 0.04)
All Values	0.03 (0.01, 0.05)	0.28 (0.13, 0.43)	0.966 (0.958, 0.973)	0.04 (0.04, 0.04)
**PF (N)**				
Below Median	63.05 (30.0, 96.1)	0.40 (0.19, 0.61)	0.948 (0.930, 0.961)	69.3 (64.6, 77.4)
Above Median	−85.6 (−167.6, −3.6)	−0.22 (−0.42, −0.01)	0.976 (0.968, 0.982)	110.6 (103.0, 123.4)
All Values	−11.48 (−74.4, 51.4)	−0.03 (−0.17, 0.12)	0.979 (0.974, 0.983)	101.5 (94.5, 109.5)
**AF (N)**				
Below Median	38.5 (18.4, 58.6)	0.40 (0.19, 0.61)	0.911 (0.881, 0.934)	48.5 (45.2, 54.2)
Above Median	20.9 (−29.9, 71.7)	0.09 (−0.12, 0.29)	0.996 (0.994, 0.997)	31.7 (29.5, 35.3)
All Values	29.7 (−8.6, 68.0)	0.11 (−0.03, 0.26)	0.992 (0.990, 0.994)	45.8 (42.6, 49.4)
**PP (W)**				
Below Median	−37.4 (−107.3, 32.5)	−0.11 (−0.32, 0.10)	0.989 (0.985, 0.992)	71.2 (66.4, 79.5)
Above Median	−93.4 (−146.3, −40.5)	−0.37 (−0.58, −0.16)	0.972 (0.962, 0.979)	83.9 (78.1, 93.6)
All Values	−65.5 (−138.9, 8.0)	−0.13 (−0.28, 0.02)	0.993 (0.992, 0.994)	80.3 (74.8, 86.7)
**AP (W)**				
Below Median	77.9 (48.2, 107.7)	0.55 (0.34, 0.77)	0.927 (0.901, 0.946)	60.2 (56.1, 67.4)
Above Median	73.2 (35.8, 110.6)	0.41 (0.20, 0.63)	0.935 (0.912, 0.952)	81.2 (75.6, 90.8)
All Values	75.6 (38.9, 112.3)	0.31 (0.16, 0.46)	0.972 (0.966, 0.978)	77.4 (72.1, 83.7)
